# A nomogram based on coagulation markers for predicting meige syndrome risk

**DOI:** 10.3389/fnagi.2025.1665276

**Published:** 2025-12-08

**Authors:** Yuanyuan Chen, Yuehua Sun, Xinyu Zhang, Yang Chen, Shuai Zhao, Mengyao Shi, Xuefeng Lv, Wenping Lian, Zhongquan Wang, Yongjun Wu, Yajie Ma, Gang Liu, Mengle Peng

**Affiliations:** 1 Department of Clinical Laboratory, The Third People’s Hospital of Henan Province, Zhengzhou, Henan, China; 2 Department of Clinical Laboratory, Center for Gene Diagnosis and Program of Clinical Laboratory, Zhongnan Hospital of Wuhan University, Wuhan, China; 3 Department of Medical Affair, The Third People’s Hospital of Henan Province, Zhengzhou, Henan, China; 4 First Clinical Medical College, Zhengzhou University, Zhengzhou, Henan, China; 5 School of Medicine, Duke University, Durham, NC, United States; 6 Department of Clinical Laboratory, The Third Affiliated Hospital of Zhengzhou University, Zhengzhou, Henan, China; 7 College of Public Health, Zhengzhou University, Zhengzhou, Henan, China; 8 Center of Meige Syndrome, The Third People’s Hospital of Henan Province, Zhengzhou, Henan, China

**Keywords:** meige syndrome, coagulation marker, nomogram, diagnosis, risk model

## Abstract

**Background:**

Meige syndrome (MS) is a rare adult-onset cranial dystonia associated with complex neuropathological mechanisms. Recent studies have shown that abnormal coagulation plays a vital role in the pathological and physiological mechanisms of neurological disease and injury. However, the association between coagulation markers and MS remains unclear.

**Methods:**

Data of 493 patients with MS and 684 healthy controls (HCs) were recruited from the Department of Clinical Laboratory of the Third People’s Hospital of Henan Province. Differences in coagulation markers were compared between different groups. Patients with MS were randomly divided into training and test cohorts. Univariate and multivariate regression analyses were used to assess independent risk factors for MS. The assumption of linearity of independent variables and the log-odds was assessed by Box-Tidwell transformation. A nomogram was constructed based on these independent risk factors. The value of the area under the receiver operating characteristic (ROC) curve (AUC), Hosmer-Lemeshow test and decision curve analysis (DCA) were used to comprehensively evaluate the performance of the model.

**Results:**

Seven coagulation markers differed significantly between the MS and HC groups. The platelet count (PLT) and plateletcrit (PCT) of MS2 patients were higher than those of MS1 patients. The activated partial thromboplastin time (APTT) was significantly elevated in patients with severe blepharospasm. Among the seven markers, APTT and fibrinogen (Fib) showed the highest diagnostic performance for MS, with AUCs of 0.7761 and 0.6464, respectively (*P* < 0.0001). Univariate and multivariate logistic regression analysis further revealed that PT%, Fib, PDW and INR were independent risk factors of MS. Based on these independent predictors, we constructed a risk prediction nomogram of MS. The ROC curve showed that the model had good discriminative performance for the diagnosis (training cohort: AUC = 0.748, 95% CI 0.713–0.782; test cohort: AUC = 0.746, 95% CI 0.697–0.795). Finally, Hosmer-Lemeshow test, calibration curves and DCA curves showed the excellent accuracy of the nomogram.

**Conclusion:**

This study provides evidence of the potential role of coagulation abnormalities in MS pathophysiology. The constructed nomogram is a quick and effective screening tool for assessing the risk of MS, thereby contributing to the diagnosis and management of MS.

## Introduction

Meige syndrome (MS) is a rare adult-onset cranial dystonia that mainly involves the muscles of the eyelid, mouth, jaw, and neck, which seriously affects the work and life of patients (Liu et al., 2021). MS commonly occurs in individuals aged between 40 and 70 years, with a notable sex predilection showing a 2–3 times higher prevalence in women than in men (Liu et al., 2022). It can be divided into three subtypes based on the craniofacial muscles involved: blepharospasm (BDS), oromandibular dystonia (ODS), and blepharospasm-oromandibular dystonia (B-ODS) (Ma H. et al., 2021). To date, the etiology and pathogenesis of MS have not been fully elucidated, and the diagnosis of MS remains challenging. The diagnosis of MS relies primarily on clinical symptoms and the exclusion of other conditions, with no established objective biomarkers available. Therefore, identifying reliable and readily accessible biomarkers for the diagnosis and risk assessment of MS patients is of great clinical significance.

MS may be linked to basal ganglia damage and nigrostriatal γ-aminobutyric acid neuron dysfunction, leading to dopamine transmitter imbalance, dopaminergic receptor hypersensitivity, and cholinergic imbalance (Tolosa and Lai, 1979). Importantly, secondary MS can occur following cerebrovascular disease, neurodegenerative diseases (e.g., Parkinson’s disease, multiple system atrophy, corticobasal ganglionic degeneration, and Huntington disease); additionally, it is associated with inflammatory mechanisms (Fu et al., 2024; LeDoux, 2009). Recent studies have reported that vascular pathology is associated with neuronal dysfunction; for instance, vascular disease can reduce blood flow and deprive neurons of essential oxygen and nutrients, which may lead to cell death and promote neurodegenerative processes (Han et al., 2020; Stanimirovic and Friedman, 2012; Watanabe et al., 2020). Parkinson’s disease (PD) is the second most common neurodegenerative disease after Alzheimer’s disease. The characteristic feature of PD is degeneration of dopamine neurons in the substantia nigra pars compacta (SNc), resulting in striatal dopamine deficiency and consequent basal ganglia dysfunction that accounts for the cardinal motor features (Albin et al., 1989; Crossman, 1987; DeLong, 1990). PD is also linked to abnormal coagulation in its pathophysiology, involving a complex interplay among neurological, vascular, and inflammatory mechanisms (Adams et al., 2019; Wang et al., 2024). Neurons communicate with astrocytes, microglia, and blood vessels, and their crosstalk at each of these interfaces affects how the neurovascular unit responds to neural activity to trigger neurovascular coupling and regulate alterations in cerebral blood flow (Attwell et al., 2010). Decreased cerebral blood flow results in disruption of blood circulation of certain brain regions (neuron, neocortex and hippocampus), leading to undersupply of the tissue, especially of oxygen (hypoxia), which can induce rapid microvascular thrombosis and fibrin deposition within the brain (Adhami et al., 2006; Li et al., 2014; Nortley et al., 2019). As a key component of the neurovascular unit, endothelial cells play critical roles in regulating vasoconstriction and dilation, the material exchange between the central nervous system (CNS) and periphery, anti-coagulation and angiogenesis (Cai et al., 2017). Under conditions of endothelial dysfunction, endothelial cells trigger fibrin formation, as well as platelet adhesion and aggregation (Yau et al., 2015). Moreover, inflammation is recognized as both a trigger and sustainer of coagulation, with extensive interplay between inflammation and coagulation involving tissue factor, thrombin, the protein C pathway, and fibrinolytic regulators (Levi and van der Poll, 2010; Wang et al., 2024). Given that MS and PD may share common pathophysiological mechanisms, we speculate that MS is associated with coagulation abnormalities. Understanding the specific characteristics of these abnormalities is crucial for improving risk stratification and guiding appropriate management strategies.

Traditional blood coagulation indicators, including platelet count (PLT), prothrombin time (PT), activated partial thromboplastin time (APTT), thrombin time (TT), fibrinogen (Fib), and international normalized ratio (INR), hold a predominant position in clinical practice. In this study, we first investigated the association between coagulation markers and MS risk. In addition, we constructed a novel and applicable scoring system for disease diagnosis by integrating clinically relevant factors.

## Materials and methods

### MS patients and healthy controls

This was a retrospective cross-sectional case-control study. The clinical and laboratory data were collected from the case management system of the Third People’s Hospital of Henan Province between January 2022 and January 2024. The study included 493 MS patients and 684 age- and gender-matched healthy controls (HCs). Patients were eligible for inclusion if they were diagnosed with MS according to clinical diagnostic characteristics. Participants were excluded if they: (1) were taking anticoagulants or antiplatelet drugs; (2) were receiving treatment with opioids or non-steroidal anti-inflammatory drugs; (3) had any hematological disease, concurrent infectious disease, hyperpyrexia, severe heart disease, metabolic disorder, inflammatory disease, autoimmune disease, severe liver/kidney disease, malignant tumor; (4) had a history of recent surgery or trauma within 6 months; (5) were pregnant or using oral contraceptives or hormone replacement therapy. This study was approved by the Third People’s Hospital of Henan Province Research Ethics Committee (2024-SZSYKY-009), and adhered to the principles outlined in the Declaration of Helsinki. The written informed consent was waived because of the retrospective nature of our study.

### Laboratory analysis

All blood samples had been collected following standardized pre-analytical protocols at the time of clinical evaluation. For coagulation testing, blood was collected into vacuum tubes containing 0.109 mol/L trisodium citrate at a blood-to-citrate ratio of 9:1, and then centrifuged at 3,500 × g for 10 min at room temperature. PT, APTT, TT, Fib, PT%, and INR were tested using coagulation analyzer (Werfen ACL TOP750, United States). Blood samples for blood routine examination were collected into K2-EDTA vacuum tubes, and PLT, and PLT related indices including platelet distribution width (PDW), plateletcrit (PCT), and mean platelet volume (MPV) were detected by blood cell counter (Mindray CAL-8000, China).

### Data analysis

R software (version 4.0.1) and GraphPad Prism 9 were used for all the analyses. Normal distribution was determined using the Kolmogorov-Smirnov test. Comparisons between two independent samples were performed using the *t*-test or Mann-Whitney *U*-test. Univariate and multivariate logistic regression analyses were conducted to identify independent risk factors of MS. The assumption of linearity of independent variables and the log-odds was assessed by Box-Tidwell transformation. The area under the curve (AUC), calibration plot, Hosmer-Lemeshow test and decision curve analysis (DCA) were used to comprehensively evaluate the performance of the model. *P* < 0.05 was considered to denote statistically significant differences.

## Results

### Comparison of clinical parameters and laboratory indexes between MS group and HC group

A total of 493 patients with MS and 684 HCs were included in our analysis. The MS group included 148 (30%) male and 345 (70%) female patients, and the HC group contained 235 (34.4%) male and 449 (65.6%) female individuals. The mean age of the MS cohort was 59.55 ± 8.576 years, and the mean age of HCs was 59.89 ± 7.395 years. There were no significant differences in age or sex distribution between the two groups (all *P* > 0.05). The results are presented in Table 1.

**TABLE 1:** The clinical parameters of MS patients and HC samples.

Variables	MS (*n* = 493)	HC (*n* = 684)	*P*-value
**Gender**			
Male	148(30%)	235(34.4%)	0.112
Female	345(70%)	449(65.6%)	
Age	59.55 ± 8.576	59.89 ± 7.395	0.4651
PT	10.64 ± 1.326	10.66 ± 1.288	0.477
PT%	109.4 ± 17.10	113.5 ± 13.33	< 0.0001****
INR	0.956 ± 0.126	0.959 ± 0.115	0.6355
TT	14.37 ± 1.930	14.15 ± 2.840	0.12
APTT	33.37 ± 5.373	34.18 ± 3.590	0.0016**
Fib	3.135 ± 0.575	3.561 ± 0.697	< 0.0001****
PLT	210.4 ± 52.867	224.3 ± 54.57	< 0.0001****
PCT	0.209 ± 0.045	0.219 ± 0.049	0.0005***
PDW	16.02 ± 0.599	16.13 ± 0.366	< 0.0001****
MPV	10.13 ± 1.359	9.857 ± 1.115	< 0.0001****

**P < 0.01, ***P < 0.001, ****P < 0.0001.

**FIGURE 1:**
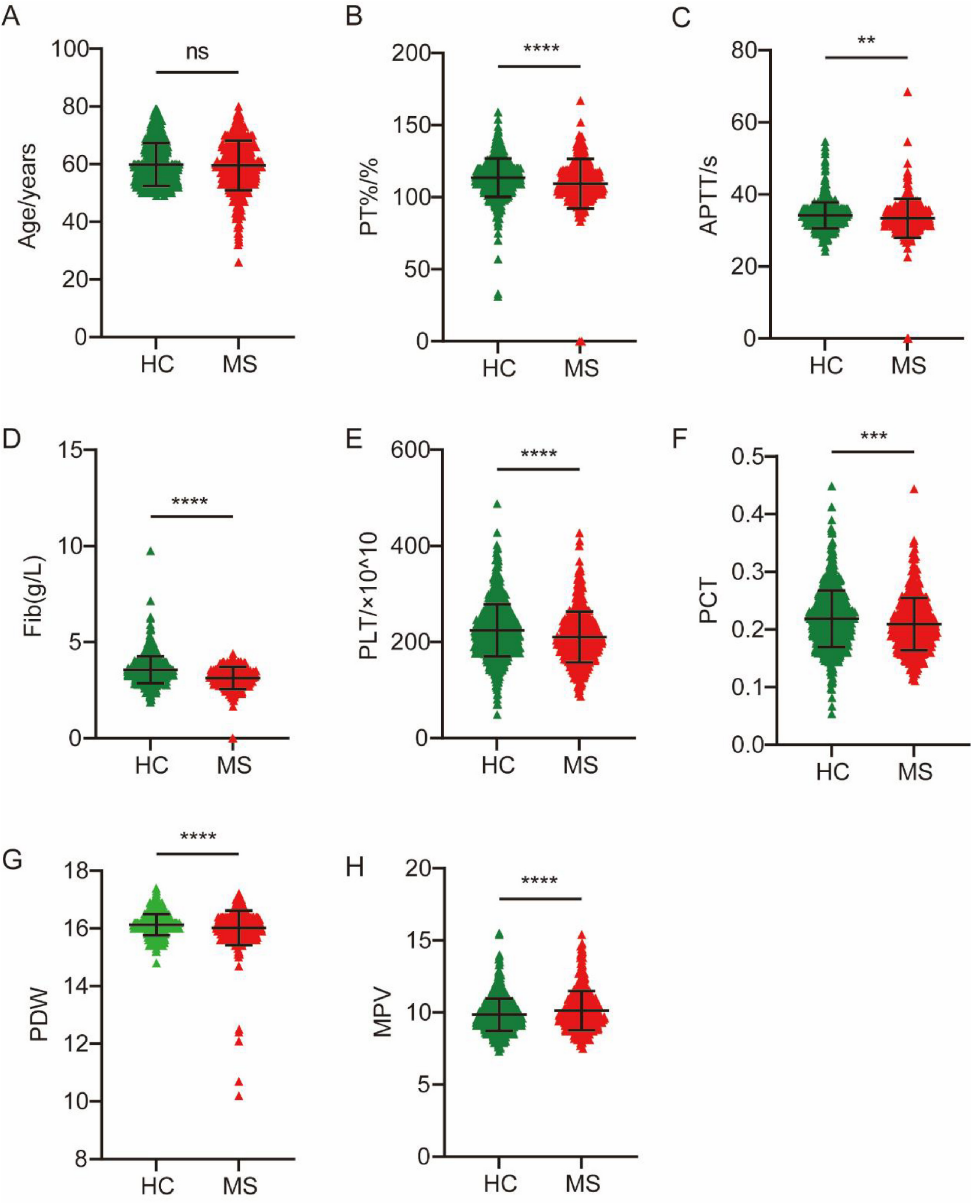
Differences in age and 7 coagulation indicators between MS and HC groups. (A) Age. (B) PT%. (C) APTT. (D) Fib. (E) PLT. (F) PCT. (G) PDW. (H) MPV. **P < 0.01, ***P < 0.001, ****P < 0.0001.

We first assessed differences in all 10 coagulation markers between the MS and HC groups. The results in Table 1 and Figure 1 show that the PT%, APTT, Fib, PLT, PCT, and PDW in the MS cohort were significantly lower than the HC cohort (MS vs. HC: PT% 109.4 ± 17.10 vs. 113.5 ± 13.33, *P* < 0.0001; APTT 33.37 ± 5.373 vs. 34.18 ± 3.590, *P* = 0.0016; Fib 3.135 ± 0.575 vs. 3.561 ± 0.697, *P* < 0.0001; PLT 210.4 ± 52.867 vs. 224.3 ± 54.57, *P* < 0.0001; PCT 0.209 ± 0.045 vs. 0.219 ± 0.049, *P* = 0.0005; PDW 16.02 ± 0.599 vs. 16.13 ± 0.366, *P* < 0.0001). Meanwhile, the MPV of the MS cohort was significantly higher than that of the HC cohort (10.13 ± 1.359 vs. 9.857 ± 1.115, *P* < 0.0001). These markers were included in the subsequent analyses.

### Different MS clinical types showed different coagulation markers levels

**FIGURE 2:**
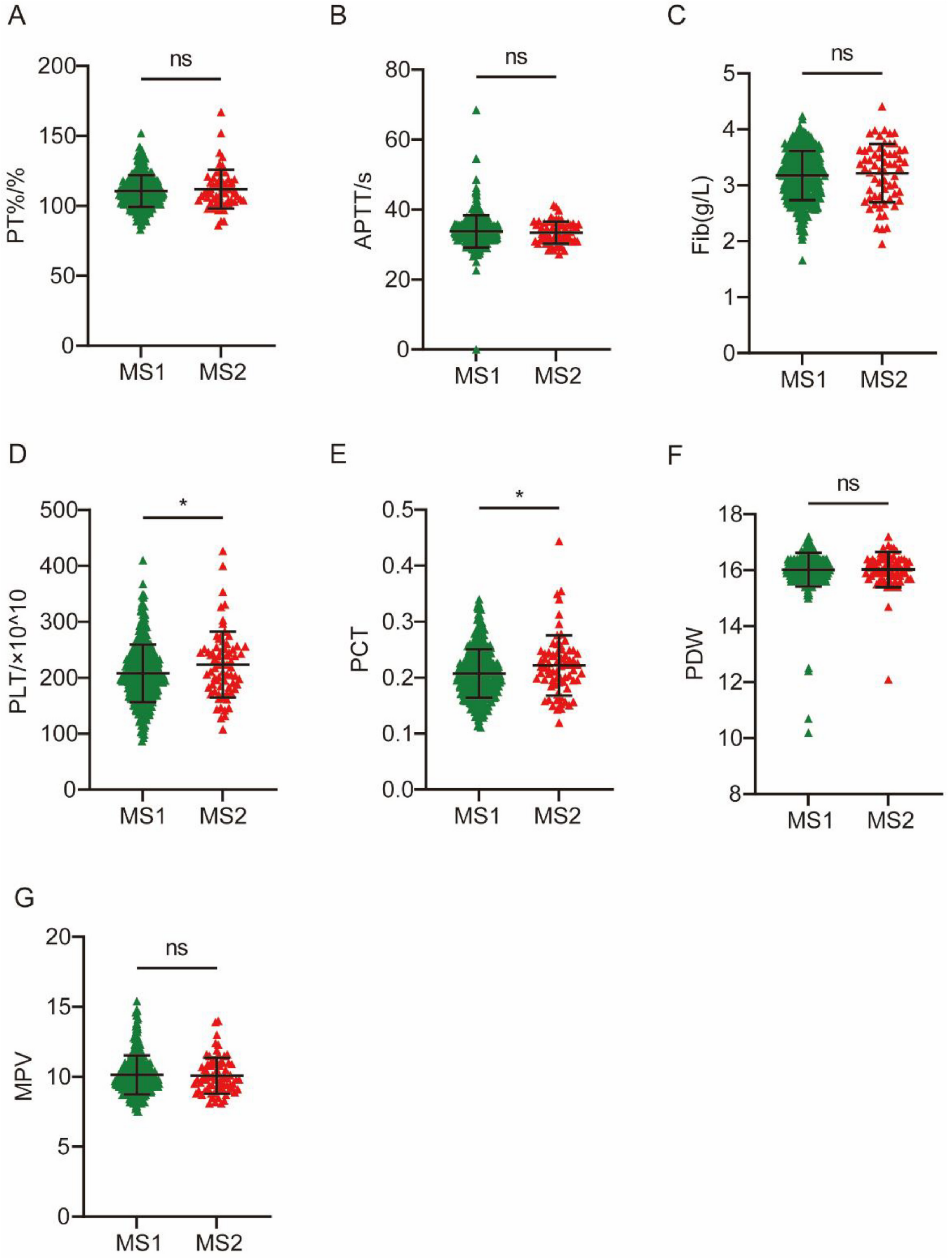
Differences in 7 coagulation indicators between MS1 and MS2-subtype patients. (A) PT%. (B) APTT. (C) Fib. (D) PLT. (E) PCT. (F) PDW. (G) MPV. *P < 0.05.

**FIGURE 3:**
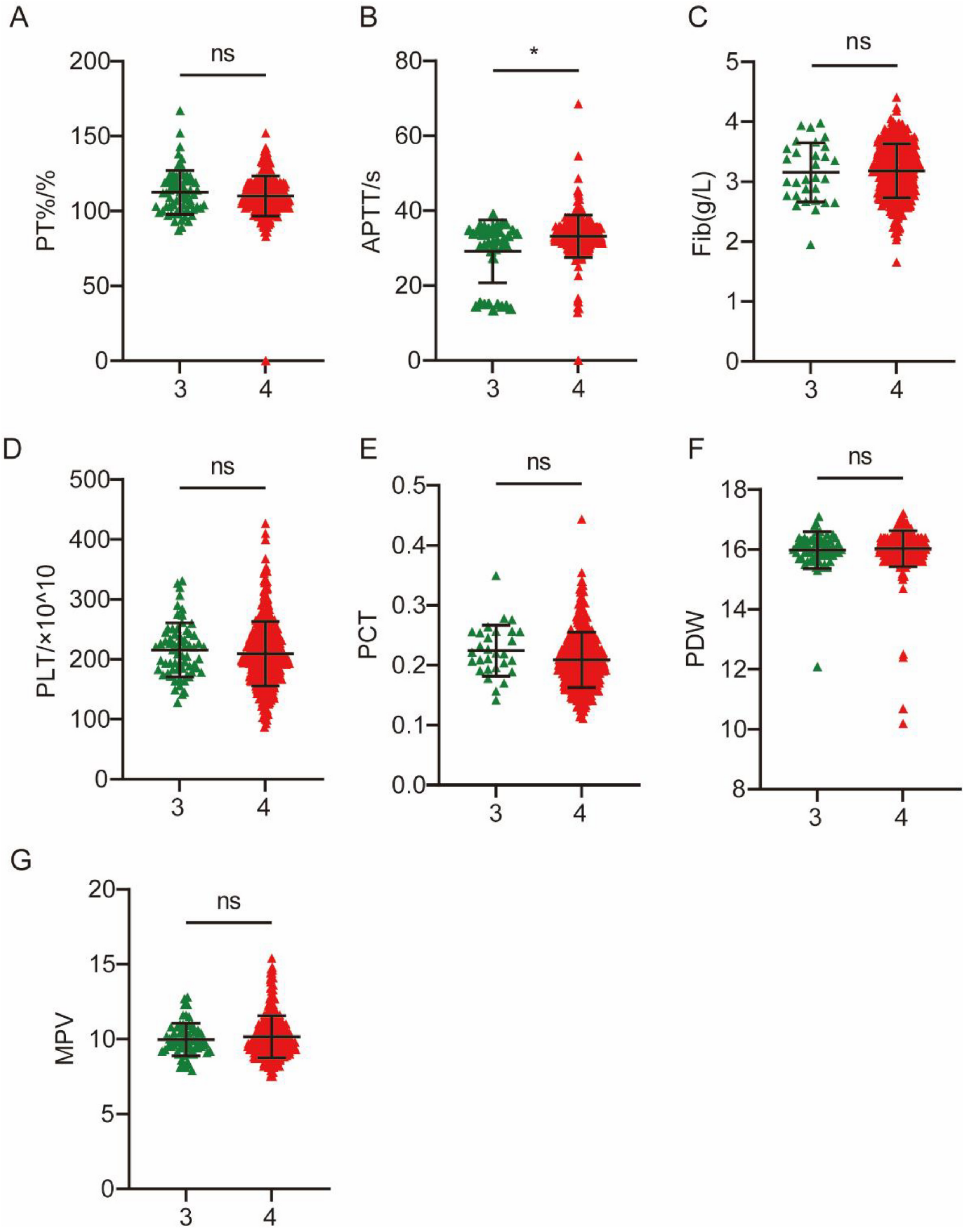
Differences in 7 coagulation indicators between Grade 3 and Grade 4 in MS patients. (A) PT%. (B) APTT. (C) Fib. (D) PLT. (E) PCT. (F) PDW. (G) MPV. *P < 0.05.

All MS patients included can be divided into two subtypes according to the involved craniofacial muscles, which we designated as MS1 and MS2. We then compared the expression of coagulation indicators between the two MS subtypes. The results showed that PLT and PCT levels of MS2 patients were higher than those of MS1 patients (Figure 2). According to the classification of blepharospasm by Shorr et al. (1985), the degree of blepharospasm can be divided into five grades. The majority of patients were divided into two grades, with 65 patients in Grade 3 and 418 patients in Grade 4. We compared coagulation markers in patients with different grades of blepharospasm. The results showed that the APTT was significantly elevated in patients with severe blepharospasm (Figure 3). These results suggest that coagulation levels can be used to distinguish between MS patients with different clinical conditions.

### Efficacy of coagulation markers in the diagnosis of MS

**FIGURE 4:**
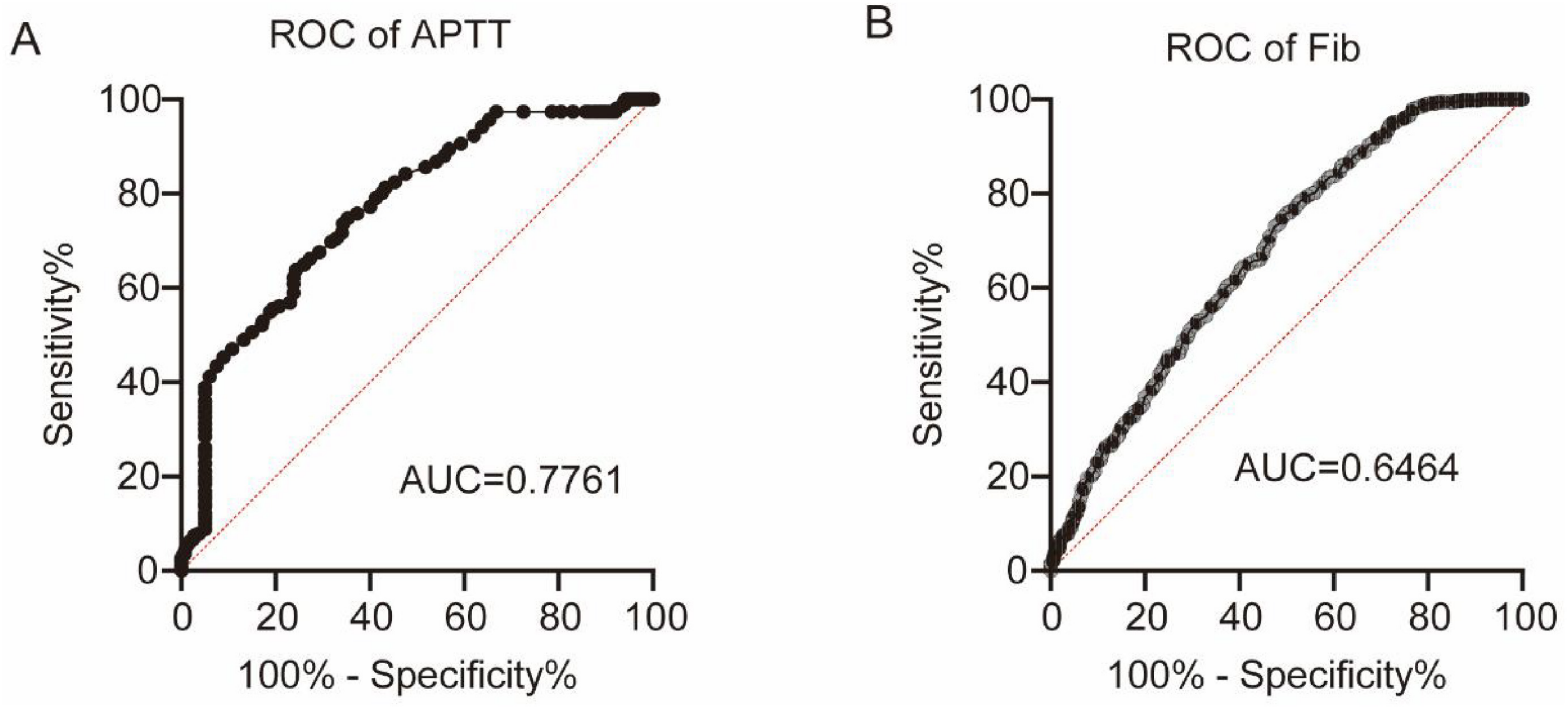
ROC curves of APTT (A) and Fib (B) for MS diagnosis.

Next, we explored the diagnostic performance of coagulation markers for MS by plotting ROC curves. Among the seven coagulation markers, APTT demonstrated the highest discriminative ability (AUC = 0.7761, *P* < 0.0001), followed by Fib (AUC = 0.6464, *P* < 0.0001), as shown in Figures 4A,B.

### Predictive model development

**FIGURE 5:**
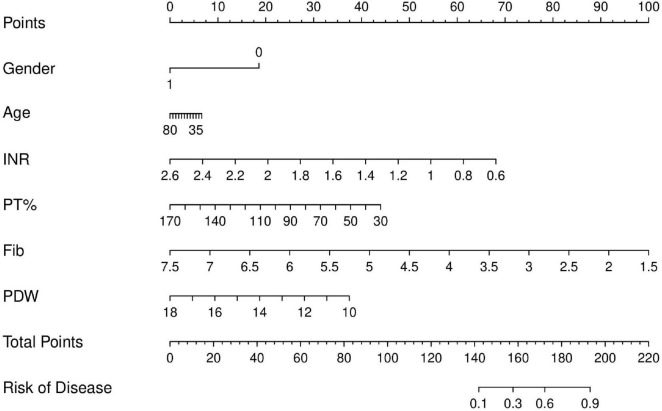
Nomogram to estimate the risk of MS based on the training cohort.

To deeply explore the role of coagulation indicators in the diagnosis of MS, we established a risk model based on the clinical parameters. All HC and MS samples were divided into training and test cohorts at a ratio of 7:3. In the training cohort, univariate logistic analysis determined five predictors, including PT%, Fib, PDW, INR, and MPV (*P* < 0.05) (Table 2). There was no evidence of nonlinear relationships between the predictors and the outcome variable (Box-Tidwell procedure, all *P* > 0.05, Supplementary Table 1). The multivariate logistic regression analysis further revealed that PT%, Fib, PDW, and INR were independent risk factors of MS (*P* < 0.05) (Table 2). Based on independent predictors obtained by multivariate logistic regression, we constructed a risk prediction nomogram of MS (Figure 5).

**TABLE 2:** Univariate and multivariate regression analyses of the training cohort.

Variables	Univariate analyses		Multivariate analyses	
	HR (95% CI)	*P*-value	HR (95% CI)	*P*-value
PT%	0.986 (0.975–0.997)	0.0173*	0.975(0.946–0.998)	0.0441*
APTT	0.978(0.945–1.011)	0.1963	0.977(0.944–1.032)	0.5783
Fib	0.308(0.230–0.408)	< 0.0001****	0.302(0.222–0.406)	< 0.0001****
PCT	0.225(0.001–4.44)	0.3282	0.0001(0.0001–169925)	0.4514
PLT	0.997(0.995–1.000)	0.0601	1.006(0.983–1.030)	0.6406
PDW	0.540(0.376–0.750)	< 0.0005***	0.167(0.053–0.207)	< 0.0001****
PT	1.085(0.946–1.279)	0.2698	0.004 (0.0001–0.3879)	0.193
INR	1.546(1.391–2.141)	0.0016**	4.108(2.627–6.893)	0.0254*
MPV	1.193(1.062–1.343)	0.0032**	2.155(0.994–3.587)	0.12

*P < 0.05, **P < 0.01, ***P < 0.001, ****P < 0.0001.

**FIGURE 6:**
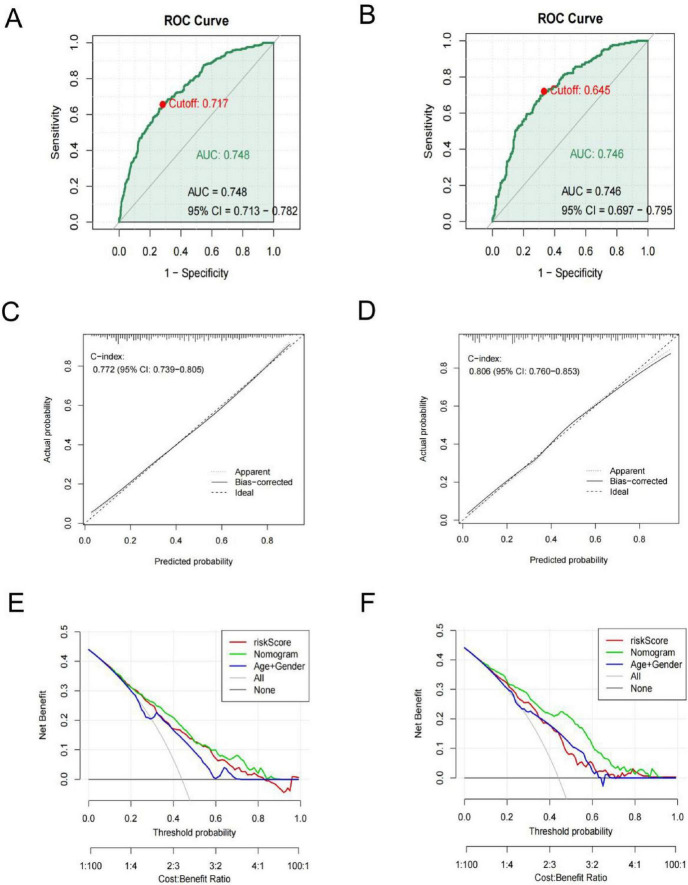
ROC curves of the nomogram for predicting MS risk in the training (A) and test (B) cohorts. Calibration plots of the training (C) and test (D) cohorts. DCA curves calibration of the training (E) and test cohorts (F).

The AUC values of the prediction model were 0.748 (95% CI 0.713–0.78 2) in the training cohort and 0.746 (95% CI 0.697–0.795) in the test cohort (Figures 6a,b), indicating good discriminative performance. The calibration plots in both cohorts showed favorable concordance between the predicted and observed probabilities (Figures 6C,D). The calibration of the nomogram was examined by the Hosmer-Lemeshow test. The result of the training set was χ^2^ = 7.929, *P* = 0.440, and that of the test cohort was χ^2^ = 13.052, *P* = 0.110. The DCA was implemented to compare the usability and advantages of the nomogram model (Figures 6E,F). The findings indicate that the nomogram demonstrated the ability to provide practical guidance for clinical diagnosis.

## Discussion

MS, a rare focal dystonic movement disorder characterized by blepharospasm and oromandibular dystonia, is associated with basal ganglia dysfunction and dopaminergic imbalance, implicating similar neural pathways as PD. Moreover, both diseases are also involved in vascular pathology and inflammation (Fu et al., 2024; Pajares et al., 2020; Park, 2016; Rektor et al., 2009), suggesting that they may share common pathophysiological mechanisms. Studies have shown that PD is also associated with dysfunctional coagulation, yet the role of coagulation abnormalities in MS remains unexplored. This study, for the first time, systematically investigated the association between coagulation profiles and MS through a substantial cohort analysis, revealing significant alterations in coagulation markers among patients with MS. This study also constructed a nomogram for MS risk prediction, showing favorable discrimination ability in both the training and test cohorts, with AUCs of 0.748 and 0.746, respectively. These findings not only expand our understanding of MS pathophysiology but also highlight the potential clinical utility of coagulation indicators in MS diagnosis and classification.

Our study found significant differences in seven coagulation markers (PT%, APTT, Fib, PLT, PCT, PDW and MPV) between MS patients and HC. Specifically, the PT% was significantly lower in the MS cohort than in the HC cohort (*P* < 0.0001), suggesting alterations in the activity of coagulation factors. APTT was lower in MS patients than in HCs, indicating alterations in the intrinsic coagulation pathway (Adamus-Grabicka et al., 2024; Berntorp and Salvagno, 2008; Chitlur, 2012; Salvagno and Berntorp, 2010). This shortened APTT can be mechanistically attributed to elevated Factor VIII activity secondary to increased von Willebrand factor levels, and both serve as established biomarkers of endothelial dysfunction and inflammation (Lelas et al., 2021; Mina et al., 2010; Radišić Biljak et al., 2024), indicating the vascular and inflammatory pathology in MS. We also found that APTT was significantly elevated in patients with severe blepharospasm, indicating the potential to distinguish between MS patients with different clinical conditions. The Fib level was significantly reduced in the MS cohort (*P* < 0.0001). Plasma Fib is involved in blood coagulation and is an important determinant of blood viscosity and hence of blood flow (Lip, 1995). A decrease in Fib levels may impair the formation and stability of blood clots, thereby increasing the risk of bleeding (Grottke et al., 2020; Li et al., 2022). In this study, AUC values for Fib and APTT in the ROC analysis highlighted their discriminatory power in distinguishing patients with MS from HCs (APTT: AUC = 0.7761, *P* < 0.0001; Fib: AUC = 0.6464, *P* < 0.0001). Furthermore, significant decreases were observed in platelet-related indicators including PLT (*P* < 0.0001), PCT (*P* = 0.0005), and PDW (*P* < 0.0001), while the MPV in the MS cohort was significantly higher than that in the HC cohort (*P* < 0.0001). In addition, our study showed that PLT and PCT of MS2 patients were higher than MS1 patients. Univariate and multivariate logistic regression analyses revealed that PT%, Fib, PDW and INR were independent risk factors for MS. PCT, PDW and MPV are a group of platelet parameters determined together in automatic complete blood count profiles. A number of molecular structures exposed to the blood stream after the disruption of endothelial cell integrity are recognized by platelets, including von Willebrand factor, collagen, laminin, vitronectin and fibrinogen (Sotnikov et al., 2013). The simultaneous decrease of both PLT and PCT indicates that platelets have been excessively consumed (Zhang et al., 2015). MPV levels increase when PLT decreases and large platelets are thought to be younger and more reactive. High MPV was associated with a variety of cardio- and cerebrovascular disorders and PD, as well as low-grade inflammatory conditions prone to arterial and venous thromboses (Braekkan et al., 2010; Gasparyan et al., 2011; Koçer et al., 2013; Ulasli et al., 2012). The seemingly contradictory coagulation profile observed in MS patients, characterized by shortened APTT alongside reduced Fib and platelet indices, likely reflects a complex dysregulation rather than a simple hyper- or hypocoagulable state. Similar complex coagulation profiles have been reported in other neurodegenerative diseases like PD, where dysfunctional coagulation is associated with involvement of neurological, vascular, and inflammatory mechanisms (Ma S. X. et al., 2021). These findings highlight the complex coagulation dysregulation underlying MS pathophysiology.

It should be noted that multiple physiological factors can influence coagulation parameters. Diet can acutely modify hemostasis: high-fat meals activate factor VII (FVIIc/FVIIa); variability in vitamin-K intake alters vitamin-K-dependent factor activation and INR stability (de Assis et al., 2009). Systemic inflammation via IL-6 up-regulates fibrinogen, and vWF/FVIII behave as positive acute-phase reactants linked to endothelial activation (Mantovani and Garlanda, 2023). Hormonal status also matters: pregnancy increases fibrinogen, FVIII, vWF, and lowers protein-S activity; combined oral contraceptives shift the profile toward hypercoagulability (Yoon, 2019). In addition, recent strenuous exercise transiently raises FVIII, vWF, thrombin-antithrombin complexes, and D-dimer with a concurrent rise in fibrinolysis, whereas habitual training tends to lower fibrinogen/PAI-1 and enhance resting tPA activity (Prisco et al., 1998).

Despite these promising findings, several limitations should be acknowledged. First, the single-center retrospective design introduced a potential selection bias and limited the generalizability of the conclusions. Validation through multicenter prospective cohorts with diverse populations is required to confirm these observations. Second, our retrospective approach could not control for these potential confounders, including preadmission pharmacotherapy, dietary habits, inflammatory status, and hormonal variations, which may have introduced unmeasured confounding and biased our effect estimates. Finally, the diagnostic complexity of MS poses inherent challenges in establishing reliable pre-disease baselines. Our analyses were restricted to post-diagnosis clinical parameters, potentially obscuring the critical temporal relationships between biomarker fluctuations and early pathological processes.

## Conclusion

In conclusion, our study provides evidence of significant differences in coagulation markers between patients with MS and HCs, highlighting the potential role of coagulation abnormalities in MS pathophysiology. The constructed nomogram is a quick and effective screening tool for assessing the risk of MS, thereby contributing to the diagnosis and management of MS. However, future studies should aim to validate these findings and explore their underlying mechanisms.

## Data Availability

The original contributions presented in the study are included in the article/Supplementary material, further inquiries can be directed to the corresponding authors.

## References

[B1] Adams B. Nunes J. M. Page M. J. Roberts T. Carr J. Nell T. A. (2019). Parkinson’s disease: A systemic inflammatory disease accompanied by bacterial inflammagens. *Front. Aging Neurosci.* 11:210. 10.3389/fnagi.2019.00210 31507404 PMC6718721

[B2] Adamus-Grabicka A. A. Hikisz P. Stepniak A. Malecka M. Paneth P. Sikora J. (2024). Molecular pro-apoptotic activities of flavanone derivatives in cyclodextrin complexes: New implications for anticancer therapy. *Int. J. Mol. Sci.* 25:8488. 10.3390/ijms25158488 39126058 PMC11312998

[B3] Adhami F. Liao G. Morozov Y. M. Schloemer A. Schmithorst V. J. Lorenz J. N. (2006). Cerebral ischemia-hypoxia induces intravascular coagulation and autophagy. *Am. J. Pathol.* 169 566–583. 10.2353/ajpath.2006.051066 16877357 PMC1780162

[B4] Albin R. L. Young A. B. Penney J. B. (1989). The functional anatomy of basal ganglia disorders. *Trends Neurosci.* 12 366–375. 10.1016/0166-2236(89)90074-x 2479133

[B5] Attwell D. Buchan A. M. Charpak S. Lauritzen M. Macvicar B. A. Newman E. A. (2010). Glial and neuronal control of brain blood flow. *Nature* 468 232–243. 10.1038/nature09613 21068832 PMC3206737

[B6] Berntorp E. Salvagno G. L. (2008). Standardization and clinical utility of thrombin-generation assays. *Semin. Thromb. Hemost.* 34 670–682. 10.1055/s-0028-1104546 19085768

[B7] Braekkan S. K. Mathiesen E. B. Njølstad I. Wilsgaard T. Størmer J. Hansen J. B. (2010). Mean platelet volume is a risk factor for venous thromboembolism: The Tromsø Study. Tromsø, Norway. *J. Thromb. Haemost.* 8 157–162. 10.1111/j.1538-7836.2009.03498.x 19496920

[B8] Cai W. Zhang K. Li P. Zhu L. Xu J. Yang B. (2017). Dysfunction of the neurovascular unit in ischemic stroke and neurodegenerative diseases: An aging effect. *Ageing Res. Rev.* 34 77–87. 10.1016/j.arr.2016.09.006 27697546 PMC5384332

[B9] Chitlur M. (2012). Challenges in the laboratory analyses of bleeding disorders. *Thromb. Res.* 130 1–6. 10.1016/j.thromres.2012.03.011 22483776

[B10] Crossman A. R. (1987). Primate models of dyskinesia: The experimental approach to the study of basal ganglia-related involuntary movement disorders. *Neuroscience* 21 1–40. 10.1016/0306-4522(87)90322-8 2955248

[B11] de Assis M. C. Rabelo E. R. Avila C. W. Polanczyk C. A. Rohde L. E. (2009). Improved oral anticoagulation after a dietary vitamin k-guided strategy: A randomized controlled trial. *Circulation* 120 1115–1122. 10.1161/CIRCULATIONAHA.109.849208 19738137

[B12] DeLong M. R. (1990). Primate models of movement disorders of basal ganglia origin. *Trends Neurosci.* 13 281–285. 10.1016/0166-2236(90)90110-v 1695404

[B13] Fu R. Lian W. Zhang B. Liu G. Feng X. Zhu Y. (2024). Development and validation of a nomogram based on inflammatory markers for risk prediction in meige syndrome patients. *J. Inflamm. Res.* 17 7721–7731. 10.2147/jir.S481649 39473982 PMC11520914

[B14] Gasparyan A. Y. Ayvazyan L. Mikhailidis D. P. Kitas G. D. (2011). Mean platelet volume: A link between thrombosis and inflammation? *Curr. Pharm. Des.* 17 47–58. 10.2174/138161211795049804 21247392

[B15] Grottke O. Mallaiah S. Karkouti K. Saner F. Haas T. (2020). Fibrinogen supplementation and its indications. *Semin. Thromb. Hemost* 46 38–49. 10.1055/s-0039-1696946 31574543

[B16] Han J. W. Maillard P. Harvey D. Fletcher E. Martinez O. Johnson D. K. (2020). Association of vascular brain injury, neurodegeneration, amyloid, and cognitive trajectory. *Neurology* 95 e2622–e2634. 10.1212/wnl.0000000000010531 32732300 PMC7713731

[B17] Koçer A. Yaman A. Niftaliyev E. Dürüyen H. Eryılmaz M. Koçer E. (2013). Assessment of platelet indices in patients with neurodegenerative diseases: Mean platelet volume was increased in patients with Parkinson’s disease. *Curr. Gerontol. Geriatr. Res.* 2013:986254. 10.1155/2013/986254 24382959 PMC3870626

[B18] LeDoux M. S. (2009). Meige syndrome: What’s in a name? *Parkinson. Relat. Disord.* 15 483–489. 10.1016/j.parkreldis.2009.04.006 19457699 PMC2743078

[B19] Lelas A. Greinix H. T. Wolff D. Eissner G. Pavletic S. Z. Pulanic D. (2021). Von willebrand factor, factor VIII, and other acute phase reactants as biomarkers of inflammation and endothelial dysfunction in chronic graft-versus-host disease. *Front. Immunol.* 12:676756. 10.3389/fimmu.2021.676756 33995421 PMC8119744

[B20] Levi M. van der Poll T. (2010). Inflammation and coagulation. *Crit. Care Med.* 38(2 Suppl.), S26–S34. 10.1097/CCM.0b013e3181c98d21 20083910

[B21] Li H. Guo Q. Inoue T. Polito V. A. Tabuchi K. Hammer R. E. (2014). Vascular and parenchymal amyloid pathology in an Alzheimer disease knock-in mouse model: Interplay with cerebral blood flow. *Mol. Neurodegener.* 9:28. 10.1186/1750-1326-9-28 25108425 PMC4132280

[B22] Li Y. Ding B. Y. Wang X. F. Ding Q. L. (2022). Congenital (hypo-)dysfibrinogenemia and bleeding: A systematic literature review. *Thromb. Res.* 217 36–47. 10.1016/j.thromres.2022.07.005 35853369

[B23] Lip G. Y. (1995). Fibrinogen and cardiovascular disorders. *Qjm* 88 155–165. 10.1093/oxfordjournals.qjmed.a069041 7767665

[B24] Liu J. Li L. Li Y. Wang Q. Liu R. Ding H. (2021). Regional metabolic and network changes in Meige syndrome. *Sci. Rep.* 11:15753. 10.1038/s41598-021-95333-8 34344985 PMC8333318

[B25] Liu J. Li L. Li Y. Wang Q. Liu R. Ding H. (2022). Metabolic imaging of deep brain stimulation in meige syndrome. *Front. Aging Neurosci.* 14:848100. 10.3389/fnagi.2022.848100 35370610 PMC8968570

[B26] Ma H. Qu J. Ye L. Shu Y. Qu Q. (2021). Blepharospasm, oromandibular dystonia, and meige syndrome: Clinical and genetic update. *Front. Neurol.* 12:630221. 10.3389/fneur.2021.630221 33854473 PMC8039296

[B27] Ma S. X. Seo B. A. Kim D. Xiong Y. Kwon S. H. Brahmachari S. (2021). Complement and coagulation cascades are potentially involved in dopaminergic neurodegeneration in α-synuclein-based mouse models of Parkinson’s disease. *J. Proteome Res.* 20 3428–3443. 10.1021/acs.jproteome.0c01002 34061533 PMC8628316

[B28] Mantovani A. Garlanda C. (2023). Humoral innate immunity and acute-phase proteins. *N. Engl. J. Med.* 388 439–452. 10.1056/NEJMra2206346 36724330 PMC9912245

[B29] Mina A. Favaloro E. J. Mohammed S. Koutts J. (2010). A laboratory evaluation into the short activated partial thromboplastin time. *Blood Coagul Fibrinolysis* 21 152–157. 10.1097/MBC.0b013e3283365770 20051842

[B30] Nortley R. Korte N. Izquierdo P. Hirunpattarasilp C. Mishra A. Jaunmuktane Z. (2019). Amyloid β oligomers constrict human capillaries in Alzheimer’s disease via signaling to pericytes. *Science* 365:eaav9518. 10.1126/science.aav9518 31221773 PMC6658218

[B31] Pajares M. Rojo A. I. Manda G. Boscá L. Cuadrado A. (2020). Inflammation in Parkinson’s disease: Mechanisms and therapeutic implications. *Cells* 9:1687. 10.3390/cells9071687 32674367 PMC7408280

[B32] Park J. (2016). Movement disorders following cerebrovascular lesion in the basal ganglia circuit. *J. Mov. Disord.* 9 71–79. 10.14802/jmd.16005 27240808 PMC4886205

[B33] Prisco D. Paniccia R. Bandinelli B. Fedi S. Cellai A. P. Liotta A. A. (1998). Evaluation of clotting and fibrinolytic activation after protracted physical exercise. *Thromb. Res.* 89 73–78. 10.1016/s0049-3848(97)00293-4 9630310

[B34] Radišić Biljak V. Tomas M. Lapić I. Saračević A. (2024). Are shortened aPTT values always to be attributed only to preanalytical problems? *Diagnosis* 11 430–434. 10.1515/dx-2024-0050 38696342

[B35] Rektor I. Goldemund D. Sheardová K. Rektorová I. Michálková Z. Dufek M. (2009). Vascular pathology in patients with idiopathic Parkinson’s disease. *Parkinson. Relat. Disord.* 15 24–29. 10.1016/j.parkreldis.2008.02.007 18403246

[B36] Salvagno G. L. Berntorp E. (2010). Thrombin generation testing for monitoring hemophilia treatment: A clinical perspective. *Semin. Thromb. Hemost* 36 780–790. 10.1055/s-0030-1265295 20978999

[B37] Shorr N. Seiff S. R. Kopelman J. (1985). The use of botulinum toxin in blepharospasm. *Am. J. Ophthalmol.* 99 542–546. 10.1016/s0002-9394(14)77954-1 4003489

[B38] Sotnikov I. Veremeyko T. Starossom S. C. Barteneva N. Weiner H. L. Ponomarev E. D. (2013). Platelets recognize brain-specific glycolipid structures, respond to neurovascular damage and promote neuroinflammation. *PLoS One* 8:e58979. 10.1371/journal.pone.0058979 23555611 PMC3608633

[B39] Stanimirovic D. B. Friedman A. (2012). Pathophysiology of the neurovascular unit: Disease cause or consequence? *J. Cereb. Blood Flow Metab.* 32 1207–1221. 10.1038/jcbfm.2012.25 22395208 PMC3390807

[B40] Tolosa E. S. Lai C. (1979). Meige disease: Striatal dopaminergic preponderance. *Neurology* 29 1126–1130. 10.1212/wnl.29.8.1126 379689

[B41] Ulasli S. S. Ozyurek B. A. Yilmaz E. B. Ulubay G. (2012). Mean platelet volume as an inflammatory marker in acute exacerbation of chronic obstructive pulmonary disease. *Pol. Arch. Med. Wewn* 122 284–290. 10.20452/pamw.1284 22576316

[B42] Wang X. Li W. Zhao X. Hu N. Wang X. Xiao X. (2024). Dysregulated coagulation in Parkinson’s disease. *Cells* 13:1874. 10.3390/cells13221874 39594622 PMC11592531

[B43] Watanabe C. Imaizumi T. Kawai H. Suda K. Honma Y. Ichihashi M. (2020). Aging of the vascular system and neural diseases. *Front. Aging Neurosci.* 12:557384. 10.3389/fnagi.2020.557384 33132896 PMC7550630

[B44] Yau J. W. Teoh H. Verma S. (2015). Endothelial cell control of thrombosis. *BMC Cardiovasc. Disord.* 15:130. 10.1186/s12872-015-0124-z 26481314 PMC4617895

[B45] Yoon H. J. (2019). Coagulation abnormalities and bleeding in pregnancy: An anesthesiologist’s perspective. *Anesth. Pain Med.* 14 371–379. 10.17085/apm.2019.14.4.371 33329765 PMC7713810

[B46] Zhang S. Cui Y. L. Diao M. Y. Chen D. C. Lin Z. F. (2015). Use of platelet indices for determining illness severity and predicting prognosis in critically Ill patients. *Chin. Med. J.* 128 2012–2018. 10.4103/0366-6999.161346 26228211 PMC4717961

